# Percolation Phase Transition of Surface Air Temperature Networks: A new test bed for El Niño/La Niña simulations

**DOI:** 10.1038/s41598-017-08767-4

**Published:** 2017-08-16

**Authors:** Lijuan Hua, Zhenghui Lu, Naiming Yuan, Lin Chen, Yongqiang Yu, Lu Wang

**Affiliations:** 1 State Key Laboratory of Severe Weather (LASW), Chinese Academy of Meteorological Sciences, Beijing, 100081 China; 2 State Key Laboratory of Numerical Modeling for Atmospheric Sciences and Geophysical Fluid Dynamics (LASG), Institute of Atmospheric Physics, Chinese Academy of Sciences, Beijing, 100029 China; 3 CAS Key Laboratory of Regional Climate Environment for Temperate East Asia, Institute of Atmospheric Physics, Chinese Academy of Sciences, 100029 Beijing, China; 4 Lab for Climate and Ocean-Atmosphere Studies, Dept. of Atmospheric and Oceanic Sciences, School of Physics, Peking University, Beijing, 100871 China; 5 International Pacific Research Center, and School of Ocean and Earth Science and Technology, University of Hawaii at Manoa, Honolulu, Hawaii USA

## Abstract

In this work, we studied the air-sea interaction over the tropical central eastern Pacific from a new perspective, climate network. The surface air temperatures over the tropical Pacific were constructed as a network, and the nodes within this network were linked if they have a similar temporal varying pattern. Using three different reanalysis datasets, we verified the percolation phase transition. That is, when the influences of El Niño/La Niña are strong enough to isolate more than 48% of the nodes, the network may abruptly be divided into many small pieces, indicating a change of the network state. This phenomenon was reproduced successfully by a coupled general circulation model, Flexible Global Ocean-Atmosphere-Land System Model Spectral Version 2, but another model, Flexible Global Ocean-Atmosphere-Land System Model Grid-point Version 2, failed. As both models have the same oceanic component, but are with different atmospheric components, the improperly used atmospheric component should be responsible for the missing of the percolation phase transition. Considering that this new phenomenon is only recently noticed, current state-of-the-art models may ignore this process and induce unrealistic simulations. Accordingly, percolation phase transition is proposed as a new test bed, which deserves more attention in the future.

## Introduction

El Niño/La Niña, characterized by large-scale anomalous temperature variation in the tropical central eastern Pacific, is one of the most prominent inter-annual modes in the earth climate system^1^. Although originated from the tropical Pacific Ocean, the sea surface temperature (SST) variability associated with El Niño/La Niña could induce significant changes in not only the tropical circulations, but could also affect the weather and climate conditions over the world^2–4^. It is intimately related to the outbreak of natural hazard events such as flood and drought^5–7^, and thus has profound socio-economic consequences. Therefore, El Niño/La Niña is one of the topic that receives significant attention by both governments and the climatology community. During the past several years, much research has been carried out to (i) understand the mechanisms of El Niño/La Niña^8–12^, and (ii) improve the prediction skills^13–15^. However, our current knowledge is still far from sufficient to perform either a long-term prediction of El Niño/La Niña events, or a reliable estimation of the El Niño/La Niña impacts.

One reason for the current research dilemma may be the lack of high-quality observations^16^. But beyond that, a more important reason should be the complex ocean-atmosphere interactions that cannot be fully addressed by current research methods. Traditionally, researchers prefer to define indexes in climate sciences, such as the well recognized El Niño/Southern Oscillation (ENSO) indicators, the Niño3.4 index, and the Southern Oscillation index (SOI)^1^. These indexes are widely used in El Niño/La Niña research, with the advantage of reducing complexity. However, this reduced complexity, on the other hand, may bring about additional disadvantages and hinder in-depth research. For example, based on the Niño3.4 index, we cannot study the internal correlations of the SSTs among different areas within the Niño3.4 region; we do not know whether the surface air temperature of different areas (grids) over the Niño3.4 region respond to the SST anomalies consistently. Moreover, we are unable to tell whether the impacts of El Niño/La Niña are different, when subjected to different internal correlation patterns of SSTs within the Niño3.4 region. These questions are all relevant to achieving a better understanding of El Niño/La Niña, but cannot be solved by analyzing simple indexes. Accordingly, new methods focusing on more detailed research regions (or even grid level) without losing too much complexity are urgently required. The use of climate network, is a type of new approach that may be productive.

Similar to the concept of complex network, in climate network different regions (e.g., grids) are considered as nodes that communicate with each other by exchanging heat, material, or even forces. The interactions between nodes are represented by links, which are quantified by measuring the similarity of time series from different nodes^17–19^. Based on the links between every two nodes, one can measure the structural changes of climate networks, as well as their responses to external forces. For example, Yamasaki *et al*. studied the effects of El Niño on climate networks of different regions, and found similar structural changes of the networks during El Niño events, even for geographical zones that are far away from the tropical central eastern Pacific^18^. Gozolchiani *et al*. analyzed the interactions between El Niño Basin and its surroundings, and found a clear autonomous behavior in the El Niño Basin, typically three months after an El Niño event begins^19^. Ludescher *et al*. calculated the link strength between the grids inside El Niño Basins and the grids outside, and found that their cooperativity tends to grow in the calendar year before an El Niño event^20^. Accordingly, a 12-mo forecasting scheme was proposed^21^. Up to now, with the development of complex network theories, the concept of climate network has become popular and many research activities have been initiated^18–23^.

Of all the current findings in complex network, percolation theory may be the most interesting^24–26^. It is based on the links of each node in a given network, but ultimately evaluates the overall state. By measuring the percentage of nodes that are isolated (no links to other nodes) from the entire network, percolation theory indicates the existence of a threshold, which, when the fraction of node removal is high enough, the considered network may convert its state, or, in other words, experience a phase transition. This theory takes into account both the complexity and the integrity of the system being studied, which is thus appropriate for climate research. In a very recent work by Lu *et al*., who applied percolation theory to the studies of El Niño/La Niña, the upper surface air temperature (SAT) network was found to experience abrupt phase transitions if the influence of El Niño/La Niña was strong enough to exceed a given threshold^27^. In this case, the connection structure was changed dramatically and the SAT network was converted into a new regime. Accordingly, effects of El Niño/La Niña were transferred to the upper SAT field, which may further affect remote regions via an atmospheric bridge. However, if there was no phase transition, the effects of El Niño/La Niña events may be limited only to local regions^27^. Therefore, from the percolation properties, one may better understand ocean-atmosphere interactions and deduce whether an El Niño/La Niña event can transfer its impact to remote regions.

However, as with many previous studies on climate network^18, 20, 22^, the findings reported by Lu *et al*. were only based on one reanalysis product^27^. It is not clear whether the conclusions still apply in multi-reanalysis datasets, let along when checking the outputs of climate models. Does the abrupt percolation phase transition universally exist independent of datasets? Can it be reproduced by coupled general circulation models (CGCMs)? These important questions have not yet been answered. In this study, using multi-reanalysis datasets, we studied the reactions of upper SAT networks to El Niño/La Niña, and verified the percolation phase transition revealed by Lu *et al*. By further comparing the performance of different models in reproducing the percolation phase transition, we proposed a new perspective for model evaluations.

The paper is organized as follows: In the “Results” section, we study the influences of El Niño/La Niña on upper sea surface air temperature networks using three different reanalysis products [National Centers for Environmental Prediction/National Center for Atmospheric Research (NCEP/NCAR) reanalysis 1, 1948–2015^28^; European Center for Medium-Range Weather Forecasts (ECMWF) Reanalysis (ERA40), 1957–2002^29^; and the Japanese 55-year Reanalysis (JRA55), 1958–2012^30^]. Next, the performance of two different CGCMs (Flexible Global Ocean-Atmosphere-Land System Model Spectral Version 2, FGOALS_s2^31^; and Flexible Global Ocean-Atmosphere-Land System Model Grid-point Version 2, FGOALS_g2^32^) are checked to see whether the percolation phase transition can be simulated correctly. By comparing the outputs of the two models, potential reasons that may lead to model simulation bias are further discussed, and a new perspective for model evaluation is proposed in the “Discussion and Conclusions” section. In the final section, we briefly describe the data, model, and methods used in this work.

## Results

**Figure 1:**
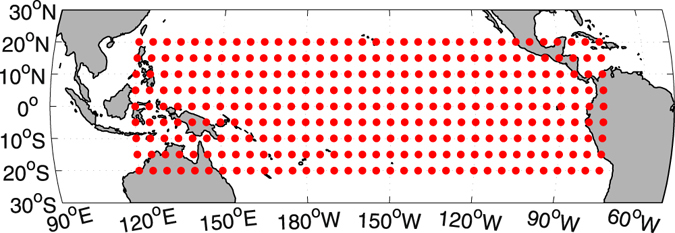
Surface air temperature network. In this study, 306 nodes with a resolution of 5° × 5° were selected and the corresponding surface air temperatures were constructed as a climate network. The figure was generated using Matlab (version R2012a, http://www.mathworks.com/pl_homepage).

In this study, the SATs over the tropical Pacific with the domains 120°E to 285°W and 20°N to 20°S were constructed as a network with a resolution of 5° × 5° (306 nodes; see Fig. 1). This SAT network has been found to be relatively independent, especially during the ENSO phase^19, 33^. Therefore, although the network has only finite-size, it is reasonable to study the percolation phase transition. To describe the network mathematically, we calculated the links between different nodes by measuring their similarity. If the link strength ${W}_{i,j}^{t}$ is stronger than a threshold *Q* (see the “Method” subsection of “Data, Models, and Methods”), we recognize that the two nodes *i* and *j* are connected at time *t* ^17^. Accordingly, the following two quantities were defined to measure the influences of El Niño/La Niña on the SAT network:

(i) The percentage of isolated nodes (*P*). If a node is not connected to any other nodes, we consider it an isolated node. The percentage of isolated nodes is thus defined as the fraction of isolated nodes over the total nodes [see Eq. (5)]^24^.
(ii) The giant component size (*S*). For some nodes of a network, if any two of them can be connected with at least one path, we consider these nodes together as a cluster. The giant component size is then defined as the ratio of the number of the nodes in the largest cluster to the number of the total connected nodes [see Eq. (6)]^24^.

By definition, *P* measures the strength of the influences by El Niño/La Niña. The higher *P* is, the more nodes are isolated from the network. *S* measures the overall properties of the network. A large *S* means that a giant cluster still spans the entire network, while a small *S* indicates that the network has been divided into many small pieces. An abrupt change of *S* from a high level to a low level indicates a change of the network state, or, in other words, a phase transition. In this work, by calculating *P* and *S*, we are able to study the reactions of the SAT network to the influences of El Niño/La Niña.

**Figure 2:**
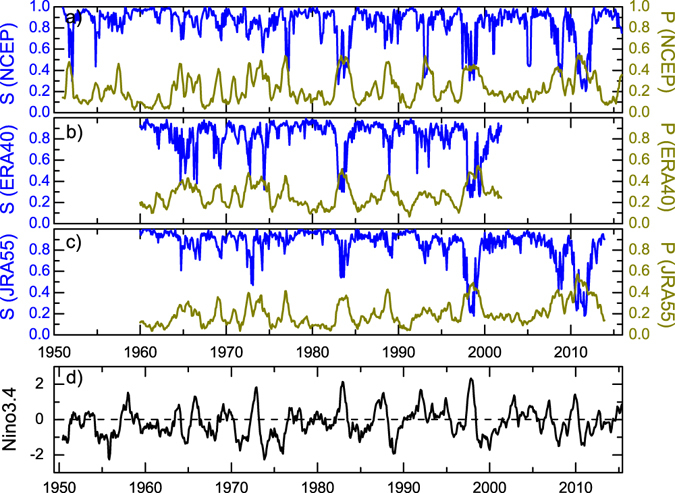
Temporal variation of the percentage of isolated nodes *P*, giant component size *S*, as well as the Niño3.4 index. (**a**–**c**) are the results calculated from the three different reanalysis datasets: (**a**) NCEP, (**b**) ERA40, and (**c**) JRA55. The yellow lines represent the percentage of isolated nodes *P* (refer to the right-hand axis), while the blue lines show the giant component size *S* (refer to the left-hand axis). (**d**) Shows the Niño3.4 index downloaded from NOAA. The x-axis in (**d**) was shifted to the right by half a year, because the *P* and *S* values were calculated using data one year before the marked time point *t* (see “Method” subsection of “Data, Model, and Methods”). As is clearly seen, all the three reanalysis datasets provide identical results. When the Niño3.4 index was larger/smaller than +0.5/−0.5, higher (lower) *P* (*S*) values were found.

First, we studied the reactions of the SAT network using three different reanalysis datasets. As shown in Fig. 2, during El Niño/La Niña events (considering that the Niño3.4 index is larger/smaller than +0.5/−0.5), the percentage of isolated nodes *P* (yellow lines) was higher than in normal periods, indicating more nodes lost their connections and became isolated under the influences of El Niño/La Niña. Correspondingly, the SAT network structure was changed with smaller *S* (blue lines). There were significant (negative) correlations between *P* and *S*, and *S* decreased abruptly when *P* was increasing. Results from all the three reanalysis datasets were in good agreement with each other, indicating identical reactions of the SAT network independent of reanalysis datasets. Since the loss of connected nodes can induce abrupt decrease of *S*, it is possible to observe a percolation phase transition in the SAT network.

**Figure 3:**
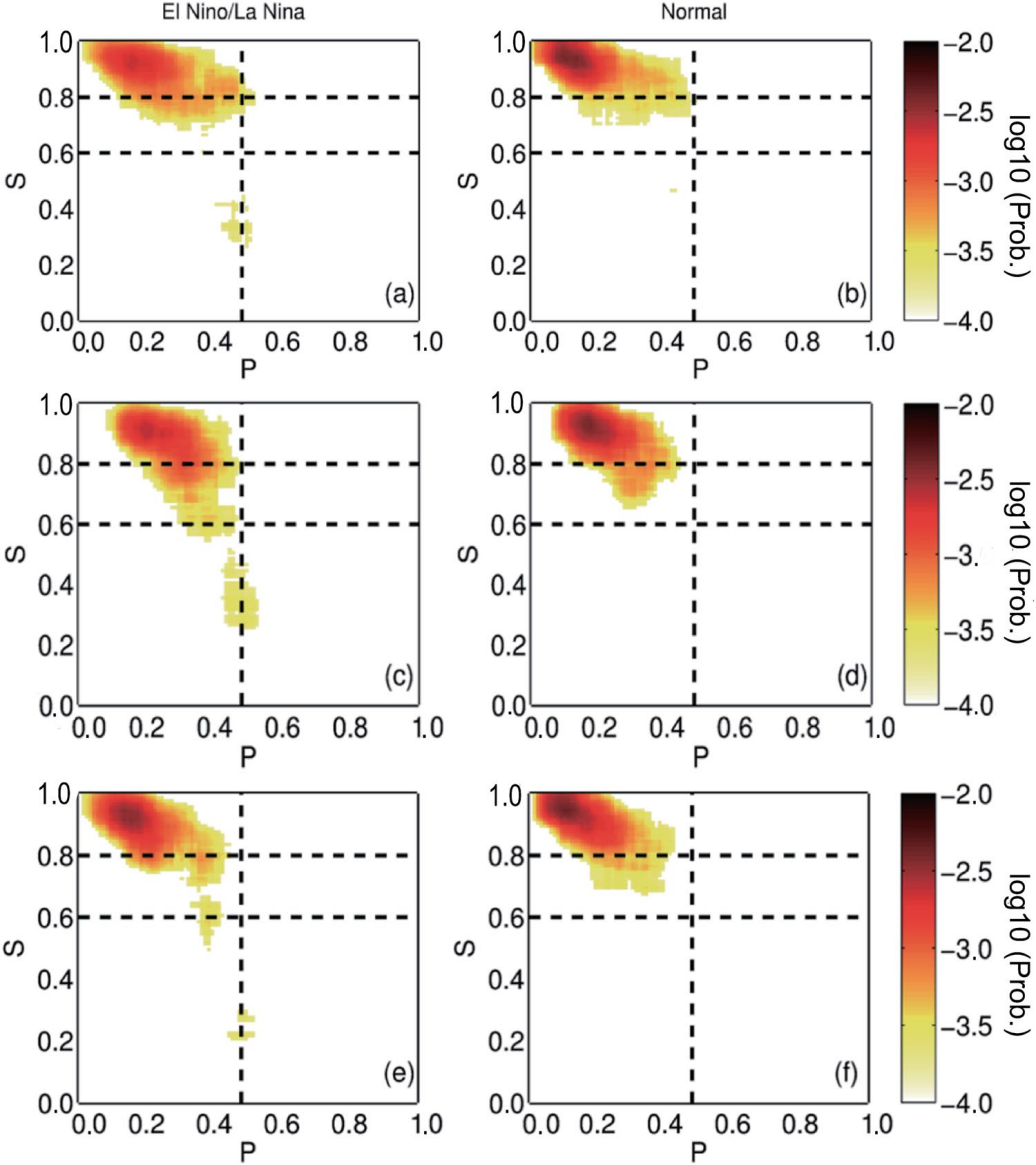
Connections of *S* and *P* in two groups. The two groups (left and right) are classified according to the Niño3.4 index. The three panels (from top to bottom) are the results of different reanalysis datasets: NCEP, ERA40, and JRA55, respectively. For normal cases, most of the *S* values are above 0.8. For the anomalous cases (El Niño/La Niña), the *S* values decreased abruptly from higher than 0.8 to lower than 0.4 at *P* = 0.48 (the vertical dashed line), indicating a percolation phase transition. The color represents the probability of having a pair of *P* and *S* at a given point of each subfigure. The numbers marked in the color bar are transformed by log10.

To check the percolation properties of the SAT network, we further classified all the considered time points into two groups and studied how the giant component size *S* varied with different values of *P*. As shown in Fig. 3b,d,f, for the “Normal” group when the Niño3.4 index was within the range [−0.5, 0.5], the *S* values obtained from the three reanalysis datasets behaved similarly, which gathered between 0.8 and 1.0 in most cases. For the “El Niño/La Niña” group when the Niño3.4 index was larger/smaller than +0.5/−0.5 [Fig. 3a,c,e], on the other hand, the *S* calculated from the three datasets all decreased abruptly from a high level (above 0.8) to a low level (below 0.6) at *P*_*k*_ = 0.48. This abrupt decrease implies that the state of the SAT network changed from a giant connected cluster to many small pieces, or in other words, from stable to unstable. *P*_*k*_ = 0.48 is a threshold that determines whether El Niño/La Niña can alter the upper level SAT network. Obviously, only when the influences of El Niño/La Niña are strong enough to isolate more than 48% of the nodes can the upper SAT network experience a phase transition. Although the three reanalysis products have different temporal coverages, they all showed identical results (Fig. 3), indicating a universal existence of the percolation phase transition.

**Figure 4:**
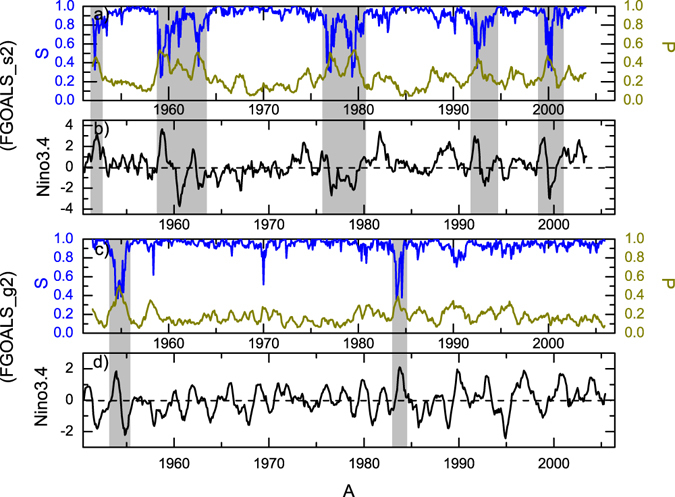
Simulated *P*, *S*, and Niño3.4 index in FGOALS_s2 and FGOALS_g2. (**a**,**b**) are the results from FGOALS_s2, while (**c**,**d**) are the results from FGOALS_g2. The gray areas highlight the cases when *P*, *S*, and the Niño3.4 index had good connections. FGOALS_s2 seems to exhibit better performance as the expected *P*/*S* reactions were simulated successfully in most cases. However, the *P* values from FGOALS_g2 seem to be underestimated. As a result, the network was unrealistically stable with high *S*.

Since the percolation phase transition were verified by multiple reanalysis datasets, it is natural to ask whether the current CGCMs can simulate this kind of phase transition. In this study, we analyzed model simulations by two CGCMs: Flexible Global Ocean-Atmosphere-Land System Model Spectral Version 2 (FGOALS_s2)^31^ and Flexible Global Ocean-Atmosphere-Land System Model Grid-point Version 2 (FGOALS_g2)^32^. Both models participated in the Coupled Model Intercomparison Project Phase 5 (CMIP5), and show excellent performance in ENSO-related behaviors among the CMIP5 models^34^. They have the same oceanic component, but different atmospheric components (see the “Model” subsection of “Data, Model, and Methods”). Therefore, the simulations are representative, especially for researching air-sea interactions. For FGOALS_s2, historical simulations from 1950 to 2003 were analyzed, while for FGOALS_g2, the time period is slightly longer, from 1950 to 2005. After constructing the network as shown in Fig. 1, we calculated the percentage of isolated nodes *P* and the giant component size *S* (Fig. 4). Generally speaking, the negative correlations between *P* and *S* were reproduced successfully by the two models, but the increase of *P* and decrease of *S* seem to be better simulated by FGOALS_s2. By comparison with the simulated Niño3.4 index, one can see remarkable reactions of *P* (or *S*) in the FGOALS_s2 simulations, and almost all the cases were well captured. For FGOALS_g2, on the other hand, the simulations were too stable. There were only few cases when significant increases (decreases) of *P* (*S*) corresponded to large anomalies of Niño3.4 in FGOALS_g2 (see the gray regions in Fig. 4). Accordingly, FGOALS_s2 seems to exhibit better performance in simulating the influences of El Niño/La Niña on the upper SAT network.

**Figure 5:**
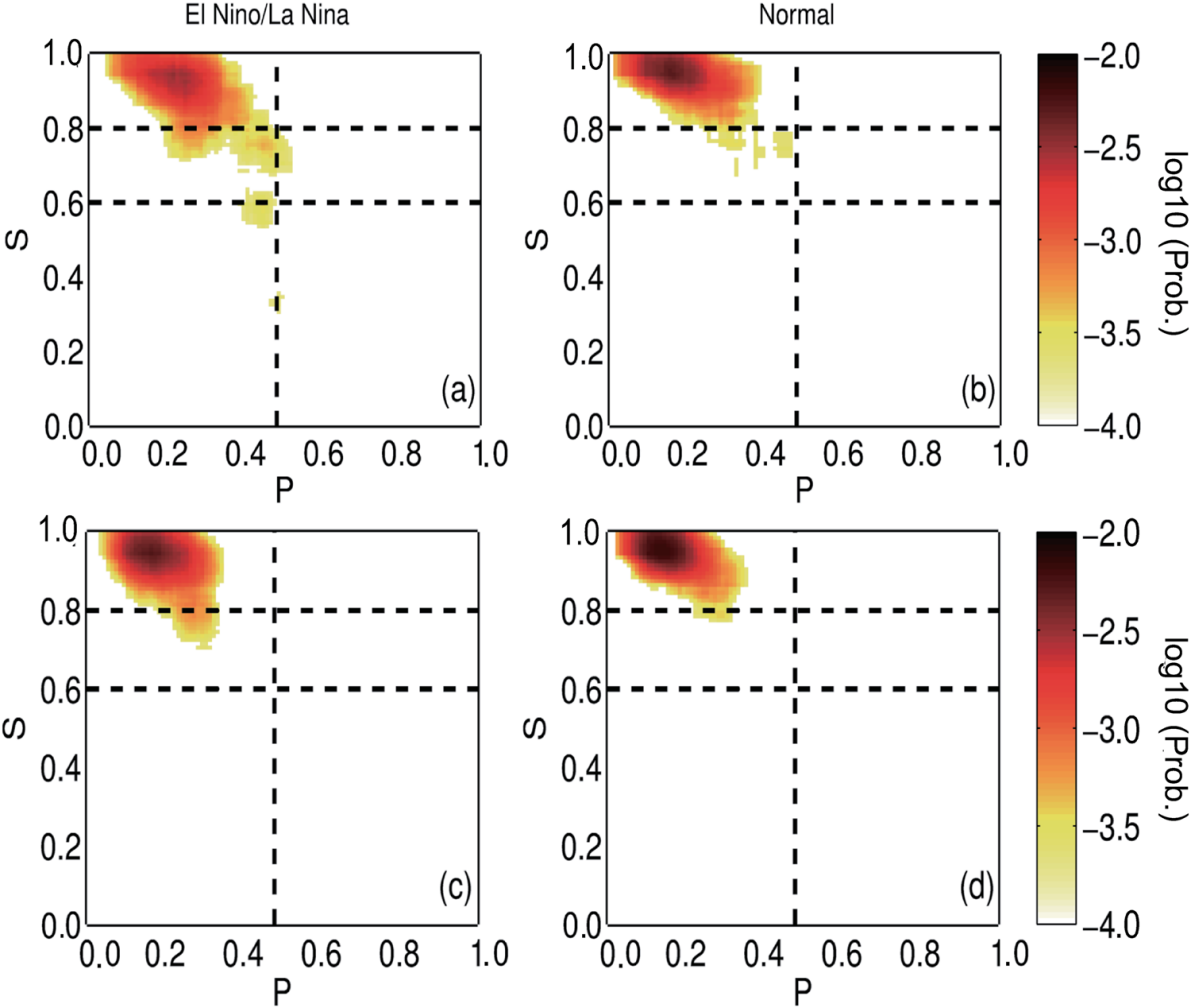
Simulated connections of *S* and *P* in two groups. Similar to Fig. 3, but showing the simulated results from FGOALS_s2 (upper panel) and FGOALS_g2 (lower panel). FGOALS_s2 exhibits better performance and reproduces the percolation phase transition successfully at *P* = 0.48 (the vertical dashed line).

Similar to Fig. 3, we further studied how the giant component size *S* varied with *P* in two groups. As shown in Fig. 5, the *S* values were above 0.8 for most cases in the “Normal” group, which was identical to the results obtained from reanalysis datasets (Fig. 3). However, when considering the “El Niño/La Niña” group, remarkable differences between the two models were revealed. The results obtained from FGOALS_s2 reproduced the abrupt percolation phase transition successfully at the correct threshold of *P*_*k*_ = 0.48, while the *S* values calculated from FGOALS_g2 still appeared above 0.8. Consequently, one cannot distinguish the two groups using the *S* behaviors, and the percolation phase transition was missed by FGOALS_g2.

**Figure 6:**
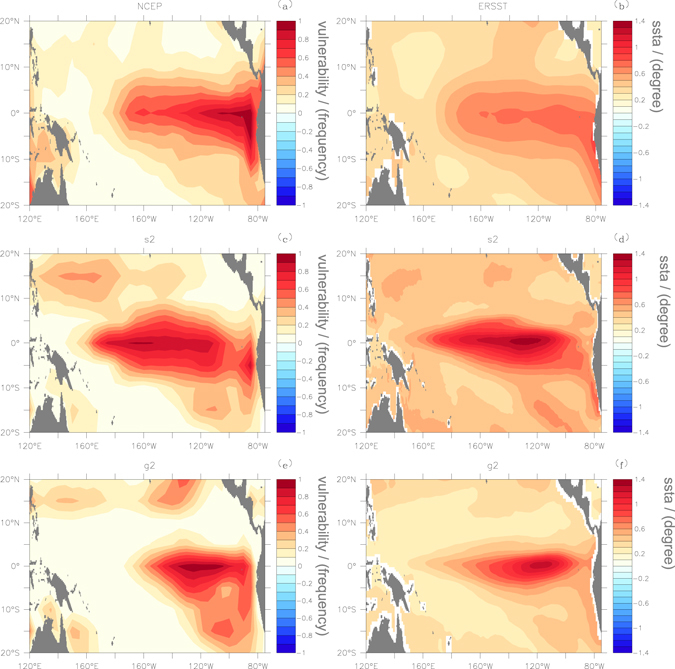
Spatial distributions of node vulnerabilities *F*_*i*_ and the sea surface temperature (SST) anomalies. Right-hand column shows the SST anomalies, which were calculated by combining both the positive anomalies and negative anomalies (taking the absolute values). Left-hand column shows the node vulnerabilities of the SAT network. The nodes with higher frequency (chance) of being isolated are marked by dark color. The upper panel represents the results from reanalysis datasets (NCAR). The middle panel shows the results of FGOALS_s2, while the bottom panel gives the simulations from FGOALS_g2. Using reanalysis datasets, one can see good agreements in the regions with strong node vulnerabilities [panel (a)] and with large SST anomalies [panel (b)]. FGOALS_s2 succeeded in reproducing this consistency, but FGOALS_g2 failed. The figure is generated using Ferret (version6.9, http://ferret.pmel.noaa.gov/Ferret/documentation/release-notes/version-6-9-release-notes).

It is worth mentioning that when analyzing the simulations we used the simulated Niño3.4 index (not the observed Niño3.4 index shown in Fig. 3) to represent the El Niño/La Niña events or normal periods. In other words, the failure in simulating the percolation phase in FGOALS_g2 is not related to the ability of the model to simulate the Niño3.4 index. In fact, many studies have pointed out that FGOALS_g2 shows excellent performance in simulating ENSO behaviors, and it is widely employed as a representative CGCM to conduct ENSO-related research^35–39^. However, regarding the ability to model air-sea interactions, especially the influences of SST anomalies on the upper surface air temperatures, FGOALS_s2 seems to exhibit better performance. To better illustrate this issue, we further studied node vulnerability *F*_*i*_ in the SAT network^27^. *F*_*i*_ is a quantity that measures how vulnerable a node is when the network is influenced. In the “Method” subsection of “Data, Models, and Methods”, it is defined as the ratio of the times that a given node was isolated to the entire time period [Eq. (7)]. If the node has a higher chance of being isolated, we consider it a node with high vulnerability. Using reanalysis datasets, we found that nodes in the network were highly vulnerable over the tropical central eastern Pacific (Fig. 6), which is the key region of El Niño/La Niña. This is reasonable as El Niño/La Niña events have the strongest influences on the upper surface air temperatures of the same region. As a result, the connections in this region are easy broken and the nodes have high chances of being isolated. From FGOALS_s2, similar results were obtained [Fig. 6c,d], and the vulnerable nodes were located in the same region that strong sea surface temperature anomalies were simulated. However, if we refer to FGOALS_g2, over the region where strong SST anomalies were simulated, only the eastern part of the network was significantly influenced with high vulnerability. In the central part, the node vulnerabilities were low. Apparently, this simulation is unrealistic, which may induce a more stable network. As shown in Fig. 4c, we indeed found small *P* values in FGOALS_g2, and for most cases the *P* values were below 0.4. Accordingly, it is natural to miss the percolation phase transition in FGOALS_g2, as the *P* values can barely reach *P*_*k*_ = 0.48.

## Discussion and Conclusion

In this work, we used three different reanalysis datasets and studied the influences of El Niño/La Niña on the upper SAT network. Identical results from the three reanalysis datasets were found, which verified the percolation phase transition of the network. That is, under the influence of El Niño/La Niña, especially when the percentage of isolated nodes *P* is higher than *P*_*k*_ = 0.48, the upper SAT network will experience an abrupt phase transition. On the other hand, if *P* is lower than *P*_*k*_, the upper SAT network will retain its main features and the effects of the SST anomalies in the tropical central eastern Pacific may be limited locally.

By analyzing the outputs of two CGCMs, we found that the percolation phase transition was successfully reproduced in FGOALS_s2, but not in FGOALS_g2. The reason for the failure in FGOALS_g2 is physically unclear, but from the node vulnerability (Fig. 6), one can speculate that FGOALS_g2 failed to fully capture the air-sea interactions in the tropical central Pacific, which further resulted in low *P* values (Fig. 4) and ultimately in missing the percolation phase transition. Since both models (FGOALS_s2 and FGOALS_g2) were designed with the identical oceanic component but coupled with different atmospheric components^31, 32^, we suggest that the contrasting performances of the two models should be attributed to the distinctive atmospheric components. Therefore, a detailed comparison between the atmospheric components in both CGCMs is highly required for further model improvement.

Our work studied the air-sea interactions over the tropical central eastern Pacific from a new perspective. By retaining the complexity in terms of a climate network, we were able to obtain more detailed information, which is important for better understanding of the underlying mechanisms of El Niño/La Niña. The percolation phase transition was found to be useful in determining whether the influences of El Niño/La Niña have been transferred upwards successfully. Meanwhile, as a verified phenomenon, it is also a good test bed for model simulations. By calculating the percentage of isolated nodes *P*, the giant component size *S*, as well as the simulated Niño3.4 index, we were able to check whether the observed percolation phase transition in the upper SAT networks can be reproduced, and further evaluated the models’ capacity to simulate air-sea interactions. Our work presented different simulations of two CGCMs, which served as typical examples that showed how to perform the model evaluation. From the good performance of FGOALS_s2, we have confidence, that the percolation phase transition can be technically well simulated. However, as a new phenomenon that has not been noticed before, even well-known models may ignore this process and fail in the simulation (see Supplementary Material, in which the results of simulations using two other models are presented). Therefore, it is important to make a detailed evaluation of the current state-of-the-art CGCMs and, if necessary, improve them from this new perspective. In followup work, we plan to perform this systematic evaluation using a large number of CGCMs.

## Data, Model, and Methods

### Data

In this study, the daily surface air temperatures from three reanalysis products were used. Data from NCEP/NCAR reanalysis 1 project (1948–2015)^28^ were downloaded from the National Oceanic & Atmospheric Administration (NOAA, provided by the NOAA/OAR/ESRL PSD, Boulder, Colorado, USA, from their website at http://www.esrl.noaa.gov/psd/data/gridded/data.ncep.reanalysis.surface.html). Data from ERA40 (1957–2002) were downloaded from the European Centre for Medium-Range Weather Forecasts^29^ (ECMWF, http://apps.ecmwf.int/datasets/data/era40-daily/levtype=sfc/). Data from JRA55 (1958–2012) were downloaded from the Japan Meteorological Agency^30^ (JMA, http://jra.kishou.go.jp/JRA-55/index_en.html#jra-55). In addition to the SATs, the Niño3.4 index was used as an indicator of El Niño/La Niña events. The index was downloaded from NOAA (http://www.esrl.noaa.gov/psd/data/climateindices/). Monthly SST anomalies were also used in this study^40^ [see Fig. 6b]. The data were also downloaded from NOAA (https://www.esrl.noaa.gov/psd/data/gridded/data.noaa.ersst.v4.html).

### Model

In this study, we analyzed simulations from two models: Flexible Global Ocean-Atmosphere-Land System Model Spectral Version 2 (FGOALS_s2)^31^ and Flexible Global Ocean-Atmosphere-Land System Model Grid-point Version 2 (FGOALS_g2)^32^. FGOALS_s2 is a state-of-the-art coupled general circulation model developed by the State Key Laboratory of Numerical Modeling for Atmospheric Sciences and Geophysical Fluid Dynamics, Institute of Atmospheric Physics (LASG/IAP). It contains atmospheric, oceanic, land and ice components. The atmospheric component is Spectral Atmospheric Circulation Model of IAP LASG, Version 2 (SAMIL2.0)^41^, with a horizontal resolution of 2.8° × 1.6°. The oceanic component is LASG/IAP Climate System Ocean Model Version 2 (LICOM2)^42^, with a horizontal resolution of about 1° × 1°. The land component is the Community Land Model CLM3^43^, and the ice component is the Community Sea Ice Model CSIM5^44^. All four components are coupled by version 6 of the NCAR coupler^44^. FGOALS_g2 is another CGCM also developed by LASG/IAP. The oceanic and land components, as well as the coupler are the same as the components used by FGOALS_s2, while the atmospheric component of FGOALS_g2 is Grid-point Atmospheric Model of IAP LASG Version 2 (GAMIL2.0)^45^, with a horizontal resolution of 2.8° × 2.8°, and the ice component is the LOS Alamos Sea Ice Model CICE4^46^.

### Methods

#### Surface air temperature Network

In this study, we employed the nonlinear synchronization measure to construct a surface air temperature network^18, 19^. For each node in Fig. 1, we first calculated anomalies by subtracting the long-term mean annual cycle (leap days are removed), *T*_*k*_(*d*), where *k* is the node index and *d* the calendar date. For every 30th day *t*, we then computed the time-delayed cross-correlation coefficients for each pair of nodes *i* and *j* over 365 days before *t*, with time lags *τ* between −200 days and 200 days. The result is denoted by ${C}_{i,j}^{t}(\tau )$. After determining the maximum, mean, and standard deviation of the absolute values of the cross-correlation coefficients ${C}_{i,j}^{t}(\tau )$ for each time point *t*, we finally defined the link strength as refs 18 and 19

1 $${W}_{i,j}^{t}=\frac{max(|{C}_{i,j}^{t}(\tau )|)-mean(|{C}_{i,j}^{t}(\tau )|)}{std(|{C}_{i,j}^{t}(\tau )|)}.$$

If the link strength is larger than a threshold *Q* (for a confidence level of 99%, *Q* = 0.57. See the following methods), we consider the two nodes as connected^17^. Otherwise, we say there is no connection. Using the Heaviside function, we can describe the connections quantitatively as ref. 18

2 $${A}_{i,j}^{t}=\theta ({W}_{i,j}^{t}-Q)=\{\begin{array}{ll}1, & {W}_{i,j}^{t} > Q\\ 0, & {W}_{i,j}^{t} < Q\end{array},$$

and the degrees of node *i* at time *t* is thus represented as ref. 27,

3 $${K}_{i}^{t}=\sum _{j=1}^{j=306}\,{A}_{i,j}^{t}.$$

If at a given time point *t*, node *i* has no connections to any other nodes, ${K}_{i}^{t}=0$, we designate it an isolated node. The isolated nodes can be counted by a new quantity, ${R}_{i}^{t}$ ^27^,

4 $${R}_{i}^{t}=\{\begin{array}{ll}1, & {K}_{i}^{t}=0\\ 0, & {K}_{i}^{t} > 0\end{array},$$

where *i* is the node index from 1 to 306.

#### Threshold Q

Using Eq. (1), one can always calculate a link strength ${W}_{i,j}^{t}$ between node *i* and node *j*, at time point *t*. To ascertain whether the link has true physical meaning, or is just a spurious result due to random effects, however, one has to determine a threshold *Q* ^18, 24^. In this study, we first shuffled the original time series at each node randomly, and calculated the link strength ${W}_{s;i,j}^{t}$ of each pair of nodes as we did for the original network. By comparing the probability density function (PDF) of link strength ${W}_{i,j}^{t}$ from the real network and the PDF of ${W}_{s;i,j}^{t}$ from the shuffled network, we can determine the threshold *Q* above which a true connection between the two nodes *i* and *j* can be confirmed. Different from Lu *et al*.^27^, we normalized the link strength before comparing the PDFs. At the significant level of 0.01, we find *Q* = 0.32.

#### Percentage of isolated nodes

In the SAT network, we consider the isolated nodes as the result of influences from El Niño/La Niña. Accordingly, we define the intensity of influences at time point *t* as refs 24 and 27,

5 $${P}^{t}=\frac{{\sum }_{i=1}^{i=306}{R}_{i}^{t}}{306},$$

where *P* ^*t*^ represents the fraction of the isolated nodes at time point *t* (see Figs 2 and 4, yellow curves).

#### Giant component size

To calculate the giant component size, one needs to find the largest cluster, where i) any two nodes can be connected with at least one path, and ii) the number of nodes is the highest. The giant component size at time point *t* is then defined as refs 24 and 27,

6 $${S}^{t}=\frac{{N}_{LC}}{306-{\sum }_{i=1}^{i=306}{R}_{i}^{t}},$$

where *N*_*LC*_ is the number of nodes in the largest cluster (see Figs 2 and 4, blue curves).

#### Node Vulnerability

To quantify the vulnerability of a given node under influences, we calculate^27^

7 $${F}_{i}=\frac{{\sum }_{t\in T}{R}_{i}^{t}}{L(T)},$$

where *L*(*T*) is the length of a given time period (the total time points) and *F*_*i*_ is the fraction of the time points when node *i* is isolated over the total time points (see Fig. 6).

## Electronic supplementary material

Supplementary Materials
