# Supplementary material for: Percolation Phase Transition of Surface Air Temperature Networks: A new test bed for El Niño/La Niña simulations
Source: Sci Rep. 2017 Aug 16;7:8324. doi: 10.1038/s41598-017-08767-4 (PMC5559492; doi:10.1038/s41598-017-08767-4)
Supplement: Supplementary file 1 — Supplementary Materials [file 41598_2017_8767_MOESM1_ESM.pdf]

Percolation Phase Transition of Surface Air Temperature Networks: A new test bed for El Niño/La Niña simulations

(Supplementary Materials)

Lijuan Hua[1,2], Zhenghui Lu[3,4], Naiming Yuan[3]*, Lin Chen[5], Yongqiang Yu[2], Lu Wang[5]

1 State Key Laboratory of Severe Weather (LASW),

Chinese Academy of Meteorological Sciences, Beijing 100081, China

2 State Key Laboratory of Numerical Modeling for Atmospheric Sciences

and Geophysical Fluid Dynamics (LASG),Institute of Atmospheric Physics,

Chinese Academy of Sciences, Beijing 100029, China

3 CAS Key Laboratory of Regional Climate Environment for Temperate East Asia,

Institute of Atmospheric Physics, Chinese Academy of Sciences, 100029, Beijing, China

4 Lab for Climate and Ocean-Atmosphere Studies, Dept. of Atmospheric and Oceanic Sciences,

School of Physics, Peking University, Beijing, 100871, China

5 International Pacific Research Center, and School of Ocean and Earth Science and Technology,

University of Hawaii at Manoa, Honolulu, Hawaii, USA

Preliminary model evaluations based on the percolation phase transition In this supplementary material, we employed the test bed proposed in this work, and evaluated two state-of-the-art models. One model is the Model for Interdisciplinary Research on Climate - Earth System Model (MIROC-ESM) that was cooperatively developed by the University of Tokyo, the National Institute for Environmental Studies (NIES), and the Japan Agency for Marine-Earth Science and Technology (JAMSTEC). The other model is the Meteorological Research Institute-Earth System Model Version 1 (MRI-ESM1) which was developed by Meteorological Research Institute of Japan. Both models participated in the Coupled Model Intercomparison Project Phase 5 (CMIP5). Detailed descriptions of the two models can be found in [1, 2].

Similar to our main text, we construct surface air temperature (SAT) networks using the data simulated by MIROC-ESM and MRI-ESM1. For MIROC-ESM, the horizontal resolution is approximately 2.8125^o (latitude) $\times$ 2.8125^o (longitude), while for MRI-ESM1, the horizontal resolution is 1.12^o (latitude) $\times$ 1.125^o (longitude). In our calculation, to keep consistent with the analysis in the main text, the simulations from these two models were all interpolated into the resolution of $5^o \times 5^o$. Meanwhile, we only used the data from 1950 to 2005. As what we did in the main text, for each model, we calculated the percentage of isolated nodes P, the giant component size S. By combining P, S with the simulated Nino3.4 index, we are able to study whether there is a percolation phase transition. As shown in Supplementary Fig.1 and Supplementary Fig.2 (similar to Fig.5 in the main text), none of the two models succeeded in reproducing the percolation phase transition. No matter in the "Normal" groups or in the "El Niño/La Niña" groups, the simulated giant component size S stayed above 0.8 for most cases. For MRI-ESM1, all the simulated P values were smaller than 0.48. While for the MIROC-ESM, the simulated P can be higher than 0.48. But even so, the corresponding S values still rarely

*Correspondence author: Naiming Yuan, Institute of Atmospheric Physics, Chinese Academy of Sciences, 100029, Beijing, China. Email: naimingyuan@hotmail.com; Tel:+86-(0)10-82995111

dropped below 0.6, indicating the missing percolation phase transition.

The unrealistic simulations by the two models indicate, that even state-of-the-art models may fail in reproducing the percolation phase transition. To improve the ability of current models in simulating air-sea interactions, it is thus necessary to pay more attention to this phenomenon and modify the underlying physical processes. Here in the supplementary material, we only present the preliminary results of this two models. A more detailed work with systematical evaluations of a large number of models will be followed in the near future.

References

[1] Watanabe, S. et al. MIROC-ESM 2010: model description and basic results of CMIP5-20c3m experiments, *Geosci. Model Dev.*, **4**, 845-872 (2011).

[2] Yukimoto, S. et al. Meteorological Research Institute-Earth System Model v1 (MRI-ESM1)-Model Description, *Technical Report of MRI*, Ibaraki, Japan, 88pp (2011).

Figure Legends

Supplementary Figure 1: **Simulated connections of S and P in MIROC-ESM.** Similar to Fig.5 in the main text. The two groups a) and b) are classified according to the simulated Nino3.4 index. When Nino3.4 index was within the range of [-0.5, 0.5], the corresponding P and S values are grouped as “Normal Phase”. While if the Nino3.4 index was larger/smaller than +0.5/-0.5, the corresponding P and S are grouped as “ENSO Phase”. The vertical dashed line shows $P = 0.48$. The color represents the probability to have a pair of P and S at a give point. The numbers marked in the color bar are transformed by log10. For both groups, the S values stayed above 0.8 for most cases. There is no percolation phase transition.

Supplementary Figure 2: **Simulated connections of S and P in MRI-ESM1.** Similar to Supplementary Fig.1, but shows the results from MRI-ESM1. Like HadGEM2-ES, no percolation phase transition was reproduced successfully.

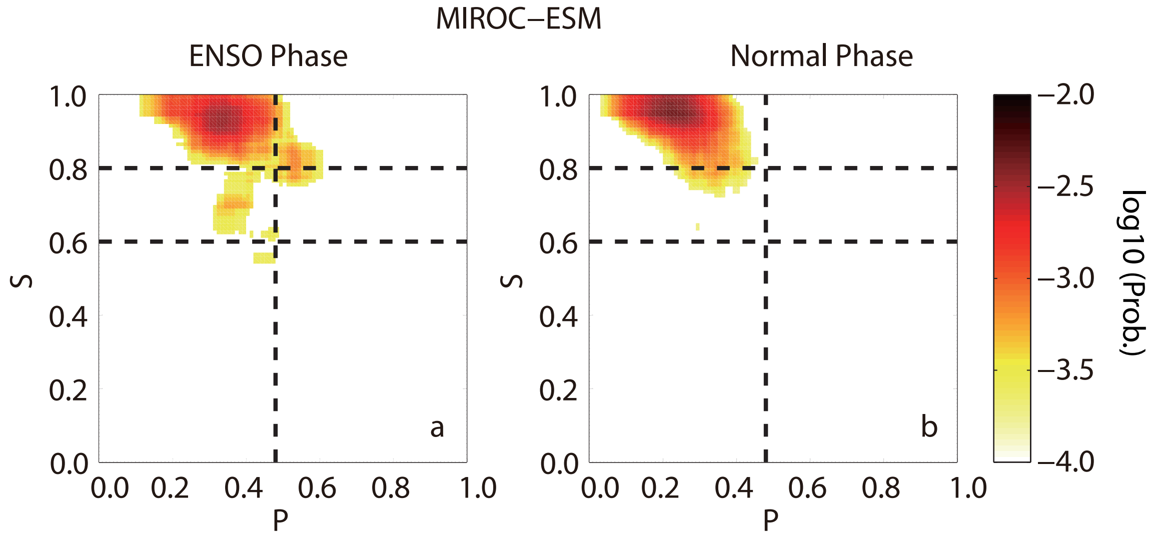

Supplementary Figure 1: **Simulated connections of S and P in MIROC-ESM.** Similar to Fig.5 in the main text. The two groups a) and b) are classified according to the simulated Nino3.4 index. When Nino3.4 index was within the range of [-0.5, 0.5], the corresponding P and S values are grouped as "Normal Phase". While if the Nino3.4 index was larger/smaller than +0.5/-0.5, the corresponding P and S are grouped as "ENSO Phase". The vertical dashed line shows $P = 0.48$. The color represents the probability to have a pair of P and S at a give point. The numbers marked in the color bar are transformed by log10. For both groups, the S values stayed above 0.8 for most cases. There is no percolation phase transition.

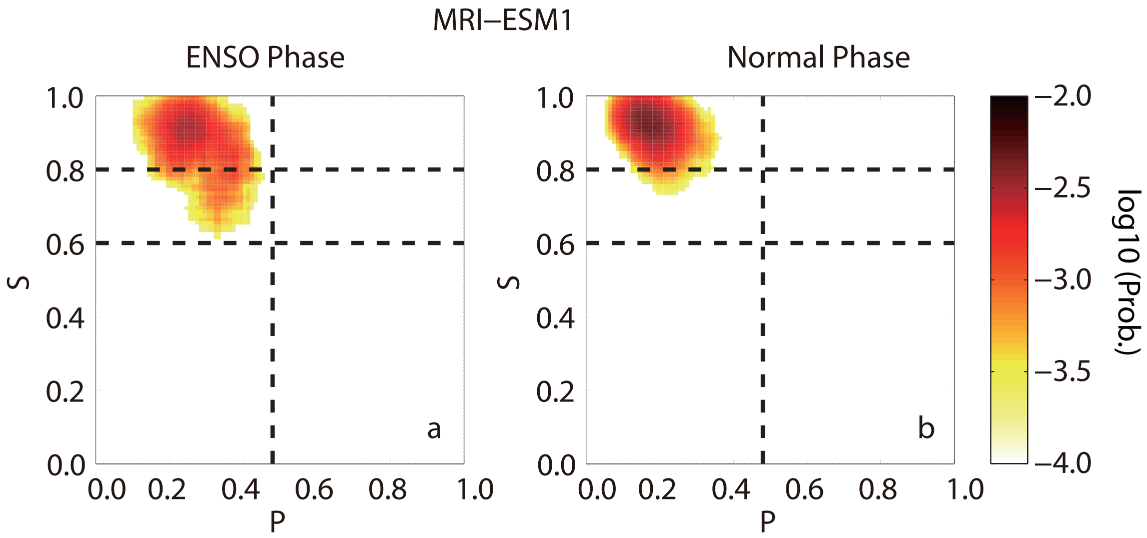

Supplementary Figure 2: **Simulated connections of S and P in MRI-ESM1.** Similar to Supplementary Fig.1, but shows the results from MRI-ESM1. Like HadGEM2-ES, no percolation phase transition was reproduced successfully.
